# The Association of Body Mass Index with Surgical Time Is Mediated by Comorbidity in Patients Undergoing Total Hip Arthroplasty

**DOI:** 10.3390/jcm10235600

**Published:** 2021-11-28

**Authors:** Francesca Cannata, Alice Laudisio, Luca Ambrosio, Gianluca Vadalà, Fabrizio Russo, Biagio Zampogna, Nicola Napoli, Rocco Papalia

**Affiliations:** 1 Department of Endocrinology and Diabetes, Campus Bio-Medico University of Rome, Via Álvaro del Portillo 200, 00128 Rome, Italy; f.cannata@unicampus.it (F.C.); n.napoli@unicampus.it (N.N.); 2 Unit of Geriatrics, Department of Medicine, Campus Bio-Medico University of Rome, Via Álvaro del Portillo 200, 00128 Rome, Italy; 3 Department of Orthopaedics and Trauma Surgery, Campus Bio-Medico University of Rome, Via Álvaro del Portillo 200, 00128 Rome, Italy; l.ambrosio@unicampus.it (L.A.); g.vadala@unicampus.it (G.V.); fabrizio.russo@unicampus.it (F.R.); b.zampogna@unicampus.it (B.Z.); r.papalia@unicampus.it (R.P.)

**Keywords:** overweight, total hip arthroplasty, total hip replacement, comorbidity, surgical time, blood loss, disease burden

## Abstract

Overweight represents a major issue in contemporary orthopaedic practice. A higher body mass index (BMI) is associated with an increase of perioperative complications following several orthopaedic procedures, in particular total hip arthroplasty (THA). However, the influence of overweight on THA surgical time is controversial. In this study, we investigated the association between BMI and surgical time analyzing the role of patients’ comorbidities. We conducted a retrospective study on 748 patients undergoing THA at our institutions between 2017 and 2018. Information regarding medical diseases was investigated and the burden of comorbidity was quantified using the Charlson score (CCI). Surgical time and blood loss were also recorded. Median surgical time was 76.5 min. Patients with surgical time above the median had both a higher BMI (28.3 vs. 27.1 kg/m^2^; *p* = 0.002); and CCI (1 vs. 0; *p* = 0.016). According to linear regression, surgical time was associated with BMI in the unadjusted model (*p* < 0.0001), after adjusting for age and sex (*p* < 0.0001), and in the multivariable model (*p* = 0.005). Furthermore, BMI was associated with increased surgical time only in patients with a Charlson score above the median, but not in others. Obesity is associated with increased surgical time during THA, especially in pluricomorbid patients, with a higher risk of perioperative complications.

## 1. Introduction

Total hip arthroplasty (THA) has been widely established as a highly successful treatment for advanced osteoarthritis (OA) of the hip, and it is largely used to improve mobility and relieve pain in ageing adults. The demand for THA has significantly increased over the years due to the proven success of these procedures to ameliorate the quality of life of patients [1]. Therefore, a significant increase in the use of THA is expected in the near future [2].

Several conditions and risk factors are responsible for this increase, among which the most important are mechanical factors, including traumatic injuries, malformations, heavy physical stress at work [3], and aging, which, in addition to favoring the onset of diseases of the musculoskeletal system, involves several comorbidities in other systems. Therefore, overweight and obesity are playing an increasingly eminent role in our society and exert a non-physiological overload that can damage the joints in the long run. 

Obesity is one of the preventable causes of death and accounts for over 2.5 million deaths annually worldwide [4]. The prevalence of overweight and obesity is also expected to progressively rise with an increasing trend in the younger population. It is assumed to determine an inevitable rise in hip OA and an exponential increase in THA procedures in this population [5]. Indeed, the higher mechanical load in the lower extremity of weight-bearing joints [6,7], metabolic disorders [8], and potential systemic mediators related to overweight and obesity might contribute to joint degeneration and favor the development of OA. In terms of clinical outcomes, overweight and obese patients seem to benefit from arthroplasty as much as non-obese patients despite a slower recovery and lower function-related scores [9,10,11,12,13]; however, obese and overweight patients have an increased risk for various short-term complications, especially infections and dislocations [14,15,16,17,18].

Nevertheless, a higher BMI is linked to an increase in perioperative complications following several orthopaedic procedures such as hip and knee arthroplasty [19,20,21,22,23,24,25], total shoulder arthroplasty [26,27], spine surgery [16,28,29,30,31,32], acetabulum fracture fixation, treatment of ankle and femur fractures [33,34,35], knee arthroscopy [36,37,38], and shoulder arthroscopy [18,39]. In addition, a higher BMI is a real risk factor for anesthesiologic and surgical practices by also lengthening the time of surgery, extending rehabilitation and convalescence, and shortening the lifespan of the prosthesis due to the overload on the operated hip. 

Based on these considerations, several studies investigated the role of preoperative BMI on the surgical time with controversial results [40,41]. The primary aim of this study was to investigate the association between BMI, comorbidities, surgical time, and length of hospitalization in patients undergoing elective primary unilateral THA. 

## 2. Materials and Methods

### 2.1. Patients

**Figure 1:**
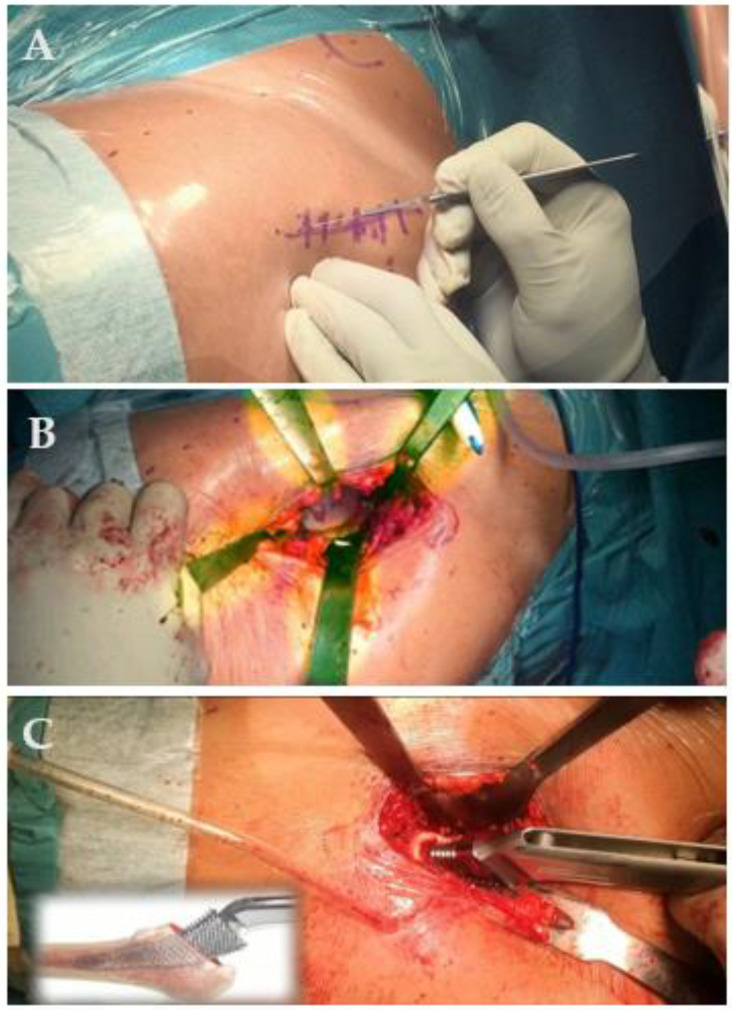
THA in overweight patients. Incision was performed using an ABMS approach (**A**). Subsequently, the acetabulum was exposed for component implantation (**B**). Eventually, the femoral canal was prepared with rasps (**C**). ABMS = anterior-based muscle sparing.

A retrospective evaluation of consecutive THAs performed at a single institution (Department of Orthopaedic and Trauma Surgery of the Campus Bio-Medico University Hospital, Rome, Italy) was performed. All data were extrapolated from a larger study. All patients upon admission to our hospital gave informed consent for the possible use of the data for scientific and not strictly clinical purposes. A consecutive series of patients with index surgery between 2017 and 2018 was analyzed. All patients affected by primary hip OA were included in the study, whereas patients suffering from secondary hip OA, femur neck fracture, and osteonecrosis of the femoral head were excluded. Clinical databases and medical records were retrospectively analyzed and then, according to the study time-lapse, inclusion and exclusion criteria, consecutive enrollment was performed. Eventually, 748 patients affected by primary hip OA were enrolled in the study. All procedures were performed by the same surgical team for prosthetics through an anterior-based muscle-sparing (ABMS) supine approach (Figure 1) [42]. A cementless standard hemispherical cup and a Corail-design cementless stem were implanted.

### 2.2. Study Variables

Electronically, medical records were reviewed, and age, gender, height, and weight, which were annotated on admission, were retrieved. BMI was calculated as weight (kg) divided by height squared (m^2^). Preoperative blood test results were reviewed in order to calculate the glomerular filtration rate (GFR) according to the Cockcroft–Gault equation. Surgical time and blood loss were extracted from operative reports. Surgical time was defined as the time from first incision to completion of wound closure. For the present analyses, surgical time was considered as continuous, as well as a dichotomous variable (above or below the median value). Blood loss was estimated using gross intraoperative visual assessment, determination of blood volume in the suction apparatus, and surgical sponges. The burden of comorbidity was calculated using the Charlson Comorbidity index (CCI), which is able to estimate the 10-year survival based on age and a history of myocardial infarction, congestive heart failure, peripheral vascular disease, stroke or transient ischemic attack, dementia, chronic obstructive pulmonary disease, connective tissue disease, peptic ulcer disease, diabetes mellitus, hemiplegia, chronic kidney disease, solid tumor, leukemia, lymphoma, and acquired immune deficiency syndrome [43]. The CCI was calculated for each patient based on perioperative information stored in medical records. Diagnoses were coded according to the International Classification of Diseases, ninth edition (ICD9).

### 2.3. Statistical Analysis

Statistical analyses were performed using IBM SPSS Statistics for Macintosh, Version 26.0 (IBM Corp., Armonk, NY, USA). Differences were considered significant at the *p* < 0.05 level. Data of continuous variables were presented as mean values ± standard deviation (SD). Median values with inter-quartile ranges were provided for non-normally distributed variables. Analysis of variance (ANOVA) for normally distributed variables was performed according to a surgical time above or below the median, as well as according to obesity; otherwise, the nonparametric Mann–Whitney U H test was adopted. The two-tailed Fisher exact test was used for dichotomous variables. The two-tailed Fisher exact test was used for dichotomous variables. Multivariable linear and logistic regression analyses were adopted to assess the association of surgical time (linear regression) and a surgical time above the median value (logistic regression) with age, sex, BMI, and all those variables which differed significantly (*p* < 0.05) in univariate analyses. Variables with abnormal distribution were analyzed after log transformation. The logistic model was also analyzed considering increasing BMI levels as underweight (<18.5 kg/m^2^), normal weight (18.5–25 kg/m^2^), overweight (25.01–30.0 kg/m^2^), obesity class I (30.01–35 kg/m^2^), obesity class II (35.01–40 kg/m^2^), and obesity class III (>40.01 kg/m^2^). The odds ratio (OR), confidence intervals (CI), and unstandardized beta (B) are shown. In addition, logistic regression analysis of the interaction terms “BMI*Charlson comorbidity score” and “BMI*sex” was performed to assess whether the association of surgical time and BMI varied according to sex and the burden of comorbidity. Additionally, the same multivariable logistic model was analyzed after stratifying for a Charlson score ≥ 1 (i.e., the median value). Eventually, Receiver Operating Characteristic (ROC) curve analysis was performed to estimate the overall predictive value and the best BMI cutoff for a surgical time above the median in patients with multimorbidity.

## 3. Results

The mean age of 748 participants was 68 ± 10 years; 413 (55%) were women. The median surgical time was 76.5 min (interquartile range 63–91 min; minimum 35 min, maximum 165 min), while the mean BMI was 27.6 ± 4.4. The median CCI was 0 (range 0–1). The main demographic characteristics of patients according to surgical time are shown in Table 1. 

Specifically, patients with a surgical time above the median had higher BMI, a more prevalent diagnosis of osteoporosis, and a higher burden of comorbidity and multimorbidity compared with other participants. Additionally, patients with longer surgical time had increased blood loss.

The characteristics of participants according to obesity are shown in Table 2.

According to linear regression, surgical time was associated with BMI in the unadjusted model (B = 1.15; 95% CI = 0.84–2.19; *p* < 0.0001), after adjusting for age and sex (B = 1.55; 95% CI = 0.87–2.22; *p* < 0.0001), and in the multivariable model (B = 0.97; 95% CI = 0.29–1.64; *p* = 0.005), adjusted for those variables which showed significant differences in univariate analyses. Likewise, in logistic regression, a surgical time above the median was associated with BMI in the unadjusted model (OR = 1.06; 95% CI = 1.02–1.09; *p* = 0.001), after adjusting for age and sex (OR = 1.06; 95% CI = 1.02–1.10; *p* = 0.001), and in the multivariable model (OR = 1.05; 95% CI = 1.01–1.10; *p* = 0.001). In a separate logistic regression model, increasing values of BMI were associated with increased probability of surgical time above the median (*p* for linear trend = 0.040). In addition, analysis of the interaction term indicated that the association of surgical time with BMI varied according to the burden of comorbidity (*p* for interaction = 0.015), but not to gender (*p* for interaction = 0.132). Additionally, after stratifying for the burden of comorbidity, BMI was associated with surgical time above the median only in patients with a CCI above the median (i.e., >1), but not in others (OR = 1.19; 95% CI = 1.04–1.37; *p* = 0.013 and OR = 1.04; 95% CI = 0.97–1.07; *p* = 0.071). Results from linear and logistic models are represented in Table 3.

**Figure 2:**
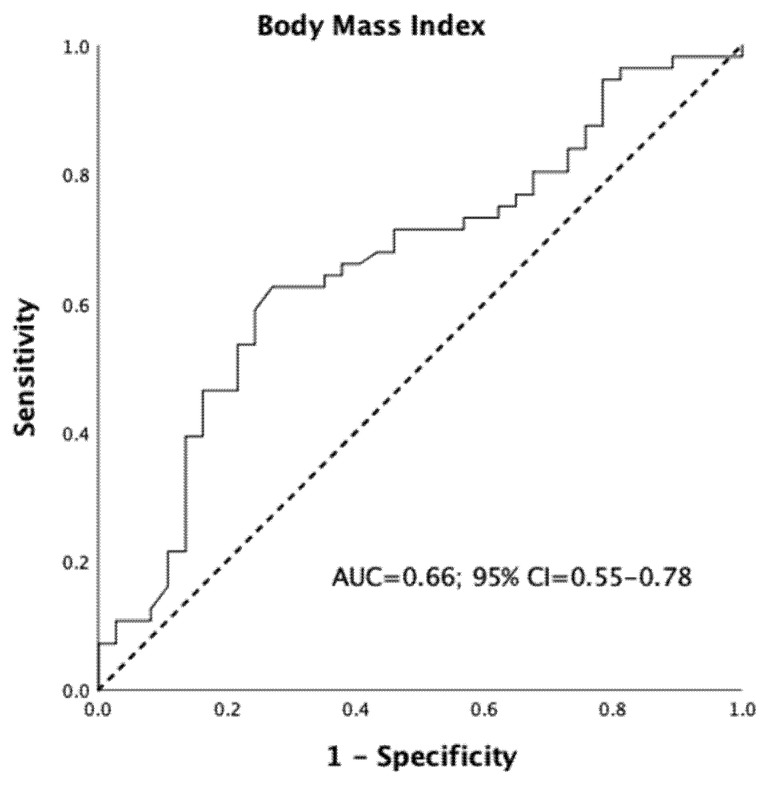
Receiver-operating characteristic (ROC) curve and corresponding area-under-the-curve (AUC) for the Body Mass Index (BMI) predicting a surgical time above the median value in patients with multimorbidity.

Eventually, in these patients, BMI was a fair predictor of a surgical time above the median (area under the curve = 0.66; 95% CI = 0.55–0.78, Figure 2). The best BMI cutoff for identifying a poor surgical time above the median was 29.2 kg/m^2^.

## 4. Discussion

In the last few decades, the prevalence of overweight and obesity has steadily increased until reaching pandemic proportions. Indeed, worldwide obesity has nearly tripled since 1975, with 1.9 billion adults being overweight in 2016 according to the World Health Organization (WHO), accounting for approximately 39% of the world population [44]. Furthermore, obesity has been recognized as the cause of 8% of global deaths in 2017, mainly due to its association with higher risks of hypertension, stroke, diabetes, and cancer [45,46].

It is widely accepted that OA is more common among obese patients due to both mechanical overloading of weight-bearing joints [47,48] and the increased secretion of pro-inflammatory cytokines [49,50], thus resulting in cartilage degradation, synovial inflammation, and osteophyte development, especially in the knee, hip, and hand joints [51]. Indeed, a study from Jiang et al. showed that each five-unit increase in BMI was associated with an 11% increase of the risk of hip OA [52]. 

THA is considered the gold standard in the treatment of end-stage hip OA. Compared to non-obese patients, overweight and obese individuals undergoing THA are usually younger and present a higher risk of perioperative complications [53]. Indeed, obesity is a multisystemic disease often accompanied by several comorbidities that may increase the likelihood of intra- or post-procedural adverse events [54]. Furthermore, several studies have demonstrated that overweight patients often present inferior post-operative outcomes following THA [55].

In this study, we found that surgical time during THA was significantly increased in patients with higher BMI and pluricomorbid conditions as calculated by the CCI. This finding may have multiple explanations. 

Generally, the surgical procedure is technically more demanding compared to normal-BMI subjects. First, patient positioning should be optimized to consent an adequate localization of anatomical landmarks and exposure of the joint, which can be difficult due to the abundancy of adipose tissue. This may lead to a reduced access to the operative field and the need for longer incisions, greater force of retraction, and higher number of retractors required to achieve an acceptable exposure [41]. Therefore, component mispositioning is more common in overweight individuals, especially at the acetabular component, which may be inadvertently over-abducted and under-anteverted [56]. In addition, great care should be devoted to safe padding of bony prominences and limb strapping, in order to reduce the risk of pressure injuries and falls from the operating table, respectively [57].

The increased burden of comorbidity may also contribute to substantially increased surgical time for several reasons. For example, patients with a history of cardiovascular or cerebrovascular events assuming low-dose aspirin for secondary prevention of subsequent accidents are at a higher risk of bleeding, which may require additional time for intraoperative hemostasis [58,59]. Similarly, while being convenient to the surgeon to reduce bleeding, intraoperative hypotension should be avoided in patients with a previous ischemic stroke due to the higher risk of cerebral hypoperfusion and recurrent ischemic events [60]. In addition, intraprocedural issues concerning airway management and alterations of vital signs in this subset of patients may occasionally require the surgeon to stop operating until the situation is under control [53]. 

Collectively, all these factors may result in increased surgical time, blood loss, and associated complications. Moreover, increased duration of patient positioning and anesthesia procedures, as well as the eventuality of intraoperative complications contribute to the increment of the overall time of occupation of the operating room. This poses both logistical and economic implications: prolonged operative duration may lead to rescheduling of programmed cases and require the operating room staff to work for longer hours [61]. As a consequence, the care of these patients demands higher costs compared to normal-BMI individuals. Indeed, it has been estimated that every five-unit increase in BMI over 30 kg/m^2^ is associated with an additional charge of $500 in hospital expenses following primary THA [62]. Furthermore, the risk of hospital readmission for revision surgery as well as the incidence of mortality, infection, and periprosthetic fractures was higher in this subset of patients [63,64].

Considering the high impact of overweight and obesity on complication rates and outcomes, weight loss is warmly encouraged before THA, as the decrease of body weight > 10% is associated with improved pain and functional scores [65]. In some cases where rapid and consistent weight loss is desirable, bariatric surgery has been employed to optimize morbidly obese patients before THA. However, a meta-analysis from Smith et al. [66] reported that preoperative bariatric surgery did not improve the risk of infection, deep venous thrombosis, pulmonary embolism, and revision surgery. Therefore, the role of bariatric surgery for managing obesity before THA surgery remains controversial. 

The present study has some limitations. First, due to its retrospective design, information bias may have occurred during the gathering of data stored in medical records, especially regarding patients’ comorbidities and calculation of the CCI. In addition, as a monocentric cohort study, selection bias relative to the homogenous characteristics of patients (mostly Caucasian and from the Rome area) may have occurred as well. However, considering that the cohort had the same diagnosis and was operated by a single surgeon using the same implant in every case, our data are quite homogenous and representative. Another interesting point of discussion is the relationship between the sole surgical time and the overall room time, intended as the time from when the patient enters the operating room to when the patient leaves the room. As discussed above, patients with higher BMIs strongly impact on operating room dynamics and logistics, with frequent positioning and anesthesia issues. Indeed, a study from Wang et al. [61] demonstrated a positive correlation between increasing BMI and higher overall room time in patients undergoing THA. Although these data were also imported from our database, they were not analyzed and will be the subject of future studies.

## 5. Conclusions

In this retrospective study, we showed that overweight patients undergoing primary THA are at a higher risk of increased surgical time and intraoperative blood loss, especially if affected by multiple comorbidities. 

## Figures and Tables

**Table 1:** Characteristics of 748 participants according to surgical time above or below the median.

	Surgical Time > Median (*n* = 347) *n* (%), Mean ± SD or IQR	Surgical Time ≤ Median (*n* = 401) *n* (%) or Mean ± SD or IQR	*p*-Value
Demographics			
Age (years)	67 (11)	68 (10)	0.220
Sex (female)	194 (47)	219 (55)	0.768
BMI	28.2 (4.3)	27.1 (4.5)	0.002
Comorbid conditions			
Diabetes	42 (10)	22 (7)	0.148
Hypertension	211 (50)	138 (39)	0.065
Dyslipidemia	97 (23)	55 (17)	0.054
Heart failure	4 (1)	2 (1)	0.704
COPD	8 (2)	7 (2)	0.798
Peripheral vascular disease	0 (0)	1 (1)	0.432
Liver failure	0 (0)	2 (1)	0.186
Renal failure	1 (1)	1 (1)	0.999
CAD	9 (2)	5 (1)	0.787
Osteoporosis	78 (66)	40 (34)	0.033
CCI	1 (0–1)	0 (0–1)	0.016
Multimorbidity	1 (0-3)	0 (0-2)	0.014
Blood tests			
Hemoglobin (g/dL)	14.0 (1.4)	14.1 (1.5)	0.830
GFR (mL/min)	107 (95–124)	96 (71–131)	0.059
Serum fasting glucose	104 (19)	105 (19)	0.649
CRP (mg/dL)	6.2 (1.5–6.6)	1.5 (1.0–5.9)	0.158
ESR (mm/h)	40 (24–82)	17 (8–23)	0.103
WBC (/μL × 10^3^)	7761 (1470)	6927 (1944)	0.344
RDW (%)	13.8 (1.5)	14.0 (2.3)	0.150
PDW (fl)	12.7 (11.5–14.3)	12.6 (11.4–13.8)	0.273
Surgery			
Estimated blood loss (mL)	300 (200–400)	200 (200–300)	<0.0001

SD = standard deviation; IQR = interquartile range; BMI = body mass index; COPD = chronic obstructive pulmonary disease; CAD = coronary artery disease; CCI = Charlson Comorbidity Index; GFR = glomerular filtration rate; CRP = C-reactive protein; ESR = erythrocyte sedimentation rate; RDW = red cell distribution width; PDW = platelet distribution width.

**Table 2:** Characteristics of 748 participants according to obesity.

	Obese Patients (*n* = 190) *n* (%) Mean ± SD or IQR	Non obese Patients (*n* = 558) *n* (%) or Mean ± SD or IQR	*p*-Value
Demographics			
Age (years)	66 (10)	69 (10)	0.005
Sex (female)	98 (52)	315 (56)	0.272
BMI	33.0 (2.6)	25.6 (3.0)	<0.0001
Comorbid conditions			
Diabetes	27 (14)	37 (7)	0.002
Hypertension	101 (53)	248 (44)	0.043
Dyslipidemia	35 (18)	117 (21)	0.531
Heart failure	2 (1)	4 (1)	0.647
COPD	6 (3)	9 (2)	0.228
Peripheral vascular disease	0 (0)	1 (1)	0.999
Liver failure	0 (0)	2 (1)	0.999
Renal failure	0 (0)	2 (1)	0.999
CAD	7 (4)	7 (2)	0.056
Osteoporosis	28 (15)	90 (16)	0.730
CCI	1 (0–1)	0 (0–1)	0.001
Multimorbidity	2 (0–3)	1 (0–2)	0.039
Blood tests			
Hemoglobin (g/dL)	14.0 (1.6)	14.0 (1.4)	0.945
GFR (mL/min)	132 (104–140)	101 (72–121)	<0.0001
Serum fasting glucose	107 (19)	103 (19)	0.012
CRP (mg/dL)	5.8 (2.0–6.6)	1.7 (1.0–5.8)	0.174
ESR (mm/h)	33 (22–75)	21 (9–45)	0.121
WBC (/mL × 10^3^)	7193 (1977)	6981 (3707)	0.455
RDW (%)	14.1 (2.3)	13.8 (1.9)	0.038
PDW (fl)	13.1 (12.7–14.8)	12.7 (11.1–13.7)	0.036
Surgery			
Estimated blood loss (mL)	300 (200–400)	250 (200–300)	0.007

SD = standard deviation; IQR = interquartile range; BMI = body mass index; COPD = chronic obstructive pulmonary disease; CAD = coronary artery disease; CCI = Charlson Comorbidity Index; GFR = glomerular filtration rate; CRP = C-reactive protein; ESR = erythrocyte sedimentation rate; RDW = red cell distribution width; PDW = platelet distribution width.

**Table 3:** Associations of surgical time above the median with the variables in the linear and logistic models.

	Linear Model			Logistic Model		
	B	95% CI	*p*-Value	OR	95% CI	*p*-Value
Age (each year)	−0.06	−0.35–0.23	0.693	1.01	0.98–1.02	0.946
Sex (female)	−0.10	−6.13–5.93	0.974	1.04	0.73–1.48	0.836
Estimated blood loss (mL)	0.09	0.07–0.11	<0.0001	1.83	1.42–2.37	<0.0001
CCI	3.58	−4.12–11.28	0.361	1.21	0.77–1.91	0.400
Osteoporosis	10.67	2.33–19.02	0.012	1.62	0.99–2.64	0.054
BMI	0.97	0.29–1.64	0.005	1.05	1.01–1.10	0.011

B = unstandardized beta; CI = confidence interval; OR = odds ratio; CCI = Charlson Comorbidity Index; BMI = body mass index.

## Data Availability

The data presented in this study are available on request from the corresponding author.
